# Stress Distribution in Immature Incisors with Regenerative Endodontic Treatment: Which Coronal Restoration Performs Best? An FEA Study

**DOI:** 10.3390/biomimetics10100674

**Published:** 2025-10-07

**Authors:** Öznur Eraslan, Mukadder İnci Başer Kolcu, Oğuz Eraslan, Sema Belli

**Affiliations:** 1 Department of Endodontics, Faculty of Dentistry, Selcuk University, Konya 42250, Türkiye; sbelli@selcuk.edu.tr; 2 Department of Medical Education and Informatics, Faculty of Medicine, Suleyman Demirel University, Isparta 32260, Türkiye; mukadderkolcu@sdu.edu.tr; 3 Department of Prosthodontics, Faculty of Dentistry, Selcuk University, Konya 42250, Türkiye; oguzeraslan@selcuk.edu.tr

**Keywords:** endocrown, endocore, immature tooth, FEA, regenerative endodontic treatment

## Abstract

**Purpose:** This study aimed to evaluate the effect of different coronal restoration methods on stresses in immature central incisors with regenerative endodontic treatment and excessive loss of coronal structure. **Methods:** A three-dimensional (3D) Finite Element Analysis (FEA) model of a maxillary central incisor treated with a 3 mm MTA coronal plug after regenerative endodontic treatment was created. Six different models were simulated: (1) intact immature tooth (control), (2) direct composite resin build-up, (3) fibre-reinforced composite build-up, (4) hybrid ceramic endocrown, (5) LiSi ceramic endocrown, and (6) endocore and ceramic crown restoration. Analyses were performed with SolidWorks/CosmosWorks, and a 150 N load was applied at a 135° angle. **Results:** Maximum tensile stresses were concentrated in the cervical region (4.577 MPa). Direct composite and fibre-reinforced restorations showed high stress in root dentin (3.891 and 3.841 MPa, respectively). The endocore/ceramic crown restoration (1.578 MPa) provided the closest stress distribution to the natural tooth (1.322 MPa). **Conclusions:** The biomechanical performance of the restoration–tooth complex depends on both the restorative material and the restoration design. In immature teeth undergoing regenerative endodontic treatment, the most biomechanically favourable restoration option was an endocore/ceramic crown.

## 1. Introduction

Immature permanent teeth with necrotic pulp present a significant clinical challenge due to their thin dentin walls and short root lengths. These structural weaknesses make teeth more susceptible to fracture and often require treatment alternatives that restore the functional and structural integrity of the tooth [1]. Regenerative Endodontic Therapy (RET) is the preferred approach for managing immature necrotic permanent teeth, owing to its ability to promote continued root development, enhance dentin wall thickness, and preserve tooth vitality [2].

The American Society of Endodontics has reported that RET is indicated for immature permanent teeth with necrotic pulp that do not require post–core in the final restoration [3]. However, many immature teeth are unsuitable for RET without sufficient coronal reinforcement because they exhibit significant coronal structure loss due to trauma or caries. In these conditions, restorations that are adhesively retained to the pulp chamber or coronal part of the root should be considered. The coronal adhesive restorations with high mechanical performance that will protect the tooth under functional loads are direct composite resin (DCR), fibre-reinforced composite resin (FRCR) [4,5], inlay/onlay restorations [6], and endocrowns [7].

DCR restorations are favoured because they have adequate aesthetic and physical properties, support strengthening tooth structures, and can be applied on-chair. However, they have disadvantages, including polymerization shrinkage, plastic wear under occlusal loads, and stress transmission to dental tissues [4]. Fibre-reinforced composite build-up restorations (FRCRs) may be a suitable option for restoring these immature teeth with significant structural loss, where root canal retention is impossible [5]. Ribbond polyethylene fibre (Ribbond, Ribbond Inc., Seattle, WA), when used as a build-up, provides retention only by adhering to the pulp chamber walls [8] and acts as a stress absorber due to its elastic modulus, enhancing fracture resistance when placed beneath the composite [5].

Endocrown restorations provide an alternative treatment option for endodontically treated teeth with significant substance loss [9]. While they offer macromechanical retention by receiving support from the pulp chamber and cavity walls, micromechanical retention is also achieved due to their cementation performance and adhesive system [10]. These restorations have been effectively applied to incisors, premolars, and molars with substantial material loss, offering a dependable option for anterior teeth following endodontic treatment because of their mechanical strength and aesthetics [11,12]. However, their success relies on the choice of material and the appropriate restoration design.

Since tooth tissue loss often affects multiple layers (enamel, dentin, or the enamel–dentin junction), restorations should mimic the natural structure through a biomimetic approach [13]. Endocrowns are preferred as biocompatible and conservative treatment options; however, their homogeneous structure may not fully replicate lost tooth biomechanics. Lithium disilicate glass–ceramic (LDGC) is a brittle and stiff material that tends to concentrate additional stress at the adhesive interface, potentially leading to adhesion failure [14]. To improve the stress distribution, researchers recommend using materials with an elastic modulus similar to dentin. Composite computer-aided design/computer-aided manufacturing (CAD/CAM) materials have become popular for their elastic modulus, wear resistance, colour integration, and ease of milling [15,16]. Creating an interlayer with glass fibre-reinforced resin under an LDGC crown to mimic natural tooth structure has been reported to reduce stresses in root canal-treated teeth [17]. A core restoration extending into the pulp chamber may be termed an “endocore,” which can be fabricated using CAD-CAM technology and adhesively cemented to root dentin, similarly to an endocrown.

Finite Element Analysis (FEA) is a reliable method for assessing the durability and mechanical performance of dental structures by simulating stresses and forces after restorative procedures [18]. It enables a detailed analysis of different materials and techniques by modelling complex geometries and loading conditions [19]. Considering the thin dentin walls and incomplete root development in immature teeth, the FEA method is an important tool to determine the impact of various restoration approaches on mechanical strength and stress distribution [20,21,22].

Previous studies generally focused on intracanal materials and root growth [21,22,23,24,25,26]; however, the effect of coronal restorations on stress distribution in teeth undergoing RET has not been sufficiently studied. The aim of this study was to evaluate how different coronal restorations affect stresses in an RET-applied maxillary incisor with coronal structure loss and incomplete root development. It was also hypothesized that different coronal treatment protocols and materials would have a similar effect on stress distribution in immature RET-treated incisors.

## 2. Materials and Methods

**Figure 1:**
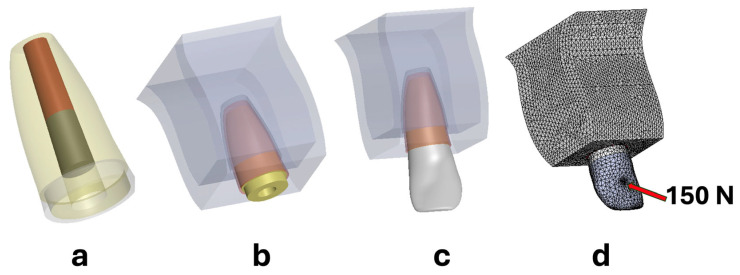
Illustration of 3D mathematical models: (**a**) transparent view of immature root with RET, (**b**) model of immature tooth with coronal structure loss and supporting structures, (**c**) main model with restoration, and (**d**) force application point and boundary conditions in the meshed model.

In this study, the SolidWorks/CosmosWorks 2018 (Dassault Systems, Waltham, MA, USA) software was used for FEA to investigate the effect of different coronal treatment procedures on the stress distribution of immature permanent maxillary central incisor teeth. The three-dimensional mathematical model simulating revascularized immature maxillary central incisor without coronal structure using MTA as a coronal plug 3 mm was modelled according to Wheeler [27] (Figure 1a–c). Total length of the simulated tooth was 16 mm. Root canal diameter was modeled as 1.28 mm at the root end, and 1.76 mm at the coronal orifice, dentin wall thickness varied from 0.3 mm at the apical to 2.3 mm at the cervical region. The tooth’s sagittal and frontal boundary lines, obtained from the atlas, were imported into the program. First, cross-sections of the structures included in the mathematical model were drawn separately for each unit in the front and right planes within the computer environment. Then, the coordinates of the contour points were entered as boundary nodes of the mathematical models. These nodes were joined to form a 3D volume for each structure, which together defined the final geometry of the FEA model. Based on the root geometry of the teeth, simplified structures were modelled, including a 0.25 mm periodontal ligament (PDL), a 0.25 mm lamina dura, a 1.5 mm cortical bone, and spongy bone [28]. Spongy bone block’s dimensions were 5.3–8.4 mm bucco-lingual width, 11 mm length, and 12.5 mm in height. The MTA coronal plug was modeled to be 3 mm long and have a diameter of 1.55 mm apically and 1.79 mm coronally.

**Figure 2:**
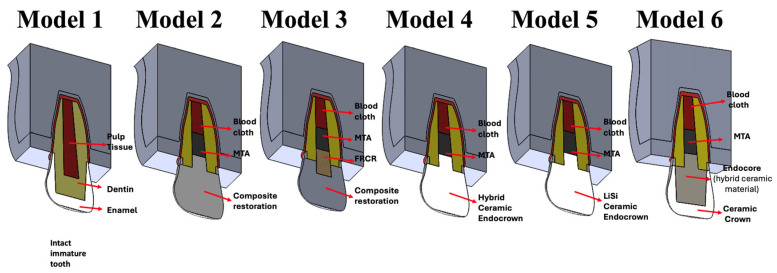
Details and structures of the FEA test models: 1. immature intact tooth, 2. DCR build-up, 3. FRCR build-up, 4. hybrid ceramic endocrown, 5. LiSi ceramic endocrown, and 6. endocore and ceramic crown.

Six different simulation models were developed as follows (Figure 2):

Model 1: The control group has an immature intact tooth.

Model 2: Coronal restoration with direct composite resin build-up.

Model 3: Coronal restoration with fibre-reinforced composite resin build-up.

Model 4: Coronal restoration with a hybrid ceramic endocrown.

Model 5: Coronal restoration with a lithium disilicate ceramic endocrown.

Model 5: Coronal restoration with an endocore and a lithium disilicate ceramic crown.

Model 2 simulated an immature maxillary incisor with coronal structure loss, restored with DCR build-up and treated with RET. In Model 3, the core structure was simulated using a polyethylene fibre (Ribbond, Seattle, WA, USA), which was wetted with a bonding agent and embedded in a flowable composite, extending to the MTA plug. The restored coronal structure was simulated by applying composite resin build-up over the created core. Model 4 was a CAD-CAM endocrown made of hybrid ceramic (Vita Enamic, VITA Zahnfabrik, H. Rauter GmbH & Co. KG, Bad Säckingen, Germany) and Model 5 simulated the use of a LiSiGC (IPS E-max, IvoclarVivadent, Schann, Liechtenstein) endocrown. In Model 6, the endocore restoration was simulated using a hybrid ceramic material (Vita Enamic, VITA Zahnfabrik, H. Rauter GmbH & Co. KG, Germany) fabricated from CAD-CAM blocks. A LiSiGC CAD-CAM crown was also simulated over the endocore restoration.

Convergence analysis was performed. The geometric models were then meshed using tetrahedral quadratic elements (Figure 1d). The total number of nodes and tetrahedral solid elements is presented in Table 1. The materials used in the study were assumed to be homogenous and isotropic. The elastic properties of the structures were acquired from the literature and manufacturers (Table 2). A static functional load of 150 N was applied to all models from the upper point of the cingulum at a 135° angle. The bottom surface of the bone structure was assumed to be fixed for boundary conditions (Figure 1d).

**Figure 3:**
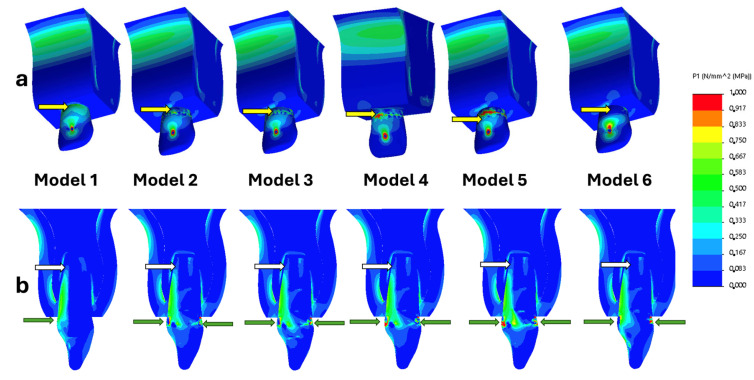
(**a**) Three-dimensional illustration of tensile stress distributions in the main models. (**b**) Bucco-palatal cross-sectional view of tensile stress distributions. Blue to red colours represent stress values from low to high, respectively.

The numerical findings were transformed into colour graphics to enhance the visualization of stress distribution within the models, presented as tensile stresses, and the scale range was limited to 0–1 MPa. The whole 3D main model, the bucco-palatal cross-section of the main model, and the bucco-palatal cross-section of dentin structure views were presented for each restoration option. The stress distributions in the models of intact immature teeth and different coronal treatment procedures are shown in Figure 3.

## 3. Results

In all models, the maximum tensile stresses were concentrated in the cervical region (Figure 3a—yellow arrows). These stresses were more concentrated in the buccal enamel structure and the palatal dentin structure (Figure 3b—green arrows). The highest tensile stresses observed in the main models were in the buccal enamel structure of the cervical region (Table 3). The root end of all models was another stress concentration area (Figure 3b—white arrows).

**Figure 4:**
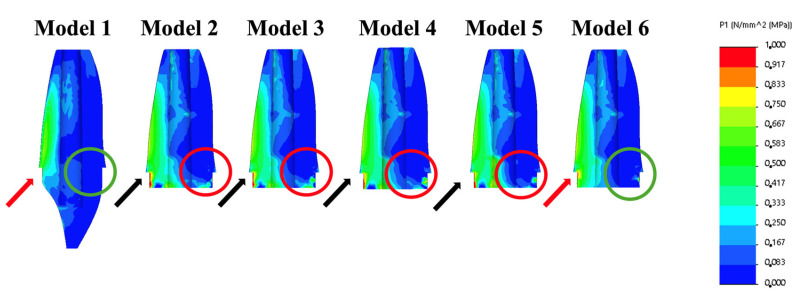
Tensile stress distributions in the dentin structures, bucco-palatal cross-sectional view. Blue to red colours represent stress values from low to high, respectively.

All five treatment options showed similar stress distributions in the test models. Both DCR and FRCR caused higher maximum tensile stress values in the root dentin structure (Figure 4—black arrows) compared to the natural tooth model. Endocore/ceramic crown restoration showed similar stress values and distributions to natural teeth (Figure 4—red arrows). The maximum tensile stress values are shown in Table 3. The highest tensile stress values observed at the dentin structure were 3.891 MPa for DCR restoration, 3.841 MPa for FRCR, 1.939 MPa for the hybrid ceramic endocrown model, 1.763 MPa for the LiSi ceramic endocrown model, 1.578 MPa for the endocore with ceramic crown restoration, and 1.322 MPa for the natural tooth model. The lowest tensile stress value (0.401 MPa) observed for the MTA plug was achieved with endocore and ceramic crown application. Endocore restoration presented similar stress distributions and values to the sound tooth model (Figure 4).

## 4. Discussion

This study provides information on the stress distribution patterns of various coronal restorations in immature maxillary central incisors treated with RET. The studied coronal restoration types had similar stress distributions but different stress values. Therefore, the hypothesis that different coronal treatment protocols and materials have identical effects on stress values in simulated RET-treated immature incisors can be rejected.

Direct composite restorations could be the simplest and most affordable restorative solution for immature teeth [29,30]. The DCR restoration model exhibited the highest tensile stress values in the dentin structure, considerably higher than those of the natural tooth. These high stress levels might be related to the plastic deformation characteristics of composite resins, influenced by their mechanical properties, and the restoration design under occlusal forces [31]. Although DCR provides aesthetic advantages and is convenient for on-chair application [29,30], its high stress levels indicate that it may not be ideal for structurally compromised immature teeth. The elastic modulus of the traditional composite resin material simulated in our study is lower than that of dentin. Furthermore, it is reported that when load is applied to multilayered material or restorations, stress concentrations are typically highest in the materials with the greatest elastic modulus [32].

Polyethylene fibres are frequently used for direct restoration of endodontically treated teeth with extreme coronal damage due to their high elastic modulus and flexibility [4,5]. A recent study observed increased fracture strength in groups using polyethylene fibres, which was attributed to the elastic modulus being comparable to that of dentin [4]. In the current study, the DCR and fibre-reinforced composite restoration models exhibited higher maximum stress values within the root dentin structure. This may be attributed to the fact that they used premolar teeth exhibiting moderate loss of coronal material that did not require occlusal crowns. In premolar teeth, the occlusal load is directed along the long axis of the tooth, while in anterior teeth, it occurs at an angle of 135 degrees to the long axis.

LiSi ceramic endocrown restorations showed lower stress values within the dentin structure than the hybrid ceramic endocrown, DCR, and FCR models. In the stress distribution figures, the LiSi ceramic endocrown restoration exhibited lower tensile stress values compared to the composite and fibre-reinforced models; however, these values were still higher than those observed in the endocore restoration model (Figure 3 and Figure 4). The lower stress values related to the endocore restoration highlight its ability to enhance the biomechanical stability of immature teeth treated with RET, suggesting that the multilayer configuration of the endocore and the ceramic crown restoration efficiently distributes occlusal forces throughout the structure of the tooth [17].

Dental ceramic materials, such as leucite ceramics, lithium disilicate, and zirconia, are commonly used in the fabrication of endocrowns [33]. Materials with an elastic modulus close to that of dentin have also started to be used for the fabrication of endocrowns because of their higher resilience [33]. Glass fibre-reinforced composites are also recommended in post–core restorations because of their flexural properties that are very similar to those of dentin [34]. Therefore, similar materials were chosen for use in this study to compare stress distribution on various restorative materials and predict clinical performance.

Gulec and Ulusoy [35] investigated different restorative materials involving feldspathic ceramics, polymer-infiltrated hybrid ceramics, and nanoceramic resins for endocrowns in maxillary first premolars. They concluded that materials that have higher elastic modulus such as feldspathic and hybrid ceramics demonstrate more tooth-friendly results. Similarly to their findings, hybrid ceramics, ceramic endocrowns, and ceramic endocore crown restorations showed lower stress values within the dentin structure in the current study.

The ceramic endocore crown restoration exhibited stress levels similar to those of natural teeth (Figure 3). This similarity can be attributed to the layered structure of the restoration, which mimics the natural tooth structure. In the model, the lost dentin tissue was represented by a hybrid ceramic layer, while the enamel tissue was simulated using ceramic. Both materials have elastic properties close to the tissues they replace. In recent years, endocrown restorations have emerged as a conservative technique for the restoration of endodontically treated teeth with coronal structure loss. With the popularity of biomimetic approaches, endocore (bilayered endocrown) restorations have begun to be tested with the aim of achieving the best performance by imitating the biomechanical properties of natural teeth. It has been reported that improved stress distributions were achievable with a bilayered restoration by using FEA [17]. Furthermore, higher fracture strength and more favourable failure patterns were reported for endocore restorations in in vitro studies [36,37]. To further investigate these design complexes, the current study aimed to investigate their impact on the longevity of restoration–tooth complexes in RET-treated immature maxillary central incisors.

This research demonstrated that all evaluated restoration techniques predominantly concentrated maximum tensile stresses in the cervical region (red) in all models, particularly in the buccal enamel and the palatal dentin, consistent with previous studies [21,23]. This region is biomechanically vulnerable due to structural transitions, making it prone to tensile forces under functional loading. While previous studies generally focused on intracanal materials and root growth [21,24], this study examined the effect of different coronal restorations on stress distribution.

The remaining dentin wall thickness in immature teeth is one of the most critical factors that directly affect stress distribution. Thin dentin walls increase stress concentration in the cervical region and increase the risk of fracture. [22]. Additionally, the depth and positioning of the MTA plug used can also play a significant role in both coronal sealing and stress transfer. Indeed, Demirel et al. reported that varying MTA plug length affects the biomechanical behavior of the root canal and that longer plugs may alter the stress distribution by reducing the volume of restorative material in the coronal region, although they strengthen the apical seal [24]. Therefore, when interpreting the modeling results clinically, it is essential to consider both the remaining dentin wall thickness and the length and position of the MTA plug in treatment planning.

This study used an immature tooth model that has not yet undergone dentin wall thickening, with a 3 mm coronal MTA plug placed according to AAE recommendations [3]. Therefore, the findings are comparable to necrotic immature teeth treated by apexification. Although apexification can create an apical barrier, it stops root growth, increases stress concentrations in the cervical region, and increases the risk of fracture [20,22,23]. In contrast, regenerative endodontic treatment (RET) promotes biomechanical durability by increasing root wall thickness in the long term. It also causes a volumetric increase in root dentin tissue and is more advantageous in preserving necrotized immature teeth [38]. Belli et al. also reported that RET provides more even stress distribution and higher fracture resistance in root dentin compared to apexification [23]. Therefore, the selection of an appropriate coronal restoration in combination with the biological advantages of RET is critical for the long-term preservation of immature teeth.

In the current study, since the structure of dentin is more sensitive to tensile stresses, which refers to the stress that causes a material to elongate or expand (in this context, the root dentin) and is an indicator of possible damage, tensile stress values were chosen for evaluation [39]. Stress concentration was notably high at the root end in all restoration models, along with the cervical area. This indicates that even with coronal reinforcement, the root end remains a crucial region for stress accumulation. Treatment strategies should focus on strengthening the crown while considering the root structure’s ability to bear stress.

Finite Element Analysis (FEA) is a commonly used method for investigating biomechanical behaviours of various restorative materials or treatment designs and predicting clinical performance [40]. FEA also allows these studies to be performed efficiently with lower costs and in shorter time [41,42]. However, FEA studies are based on various assumptions; therefore, many details are idealized, simplified, or ignored [43]. One limitation of this study was that only static loading conditions were applied; however, teeth are subjected to dynamic and variable forces such as those encountered during mastication and trauma. Additionally, the materials examined in the FEA studies were assumed to be homogeneous and isotropic. Another limitation of this study is the assumption of complete bonding of the adhesive interfaces. However, since different design models were compared and all models were assumed to have bonded interfaces, this effect is expected to be minimal. Furthermore, validation of the FEA results with experimental tests would be beneficial. Future studies should include dynamic loading and investigate the use of more advanced material models to simulate the behaviour of dental tissues.

Within the limitations of this study, it was found that the biomechanical performance of the restoration–tooth complex depends on both the restorative material and restoration design. Endocore and crown restorations may offer the most favourable biomechanical outcomes for immature teeth treated with RET, closely replicating the stress distribution observed in natural teeth. This emphasizes the potential for endocore restorations to reduce fracture risk and enhance long-term outcomes for patients with immature necrotic teeth. Further clinical research is recommended to confirm these results and investigate this restoration technique.

## Figures and Tables

**Table 1:** Number of nodes and elements in groups.

	Model No	Nodes	Elements
**Intact immature tooth**	Model 1	233,311	162,214
**Coronal composite restoration**	Model 2	232,196	156,705
**Coronal composite restoration with fiber reinforcement**	Model 3	232,202	156,712
**Hybrid ceramic endocrown**	Model 4	232,945	157,257
**LiSi endocrown**	Model 5	232,945	157,257
**Endocore and ceramic crown**	Model 6	235,127	159,112

**Table 2:** Elastic properties of the investigated structures [17].

Materials/Structure	Elastic Modulus E; MPa	Poisson’s Ratio
Enamel	84,100	0.33
Dentin	18,600	0.31
Pulp Tissue	3	0.45
Periodontal ligament	0.07	0.45
Composite Resin	16,400	0.28
Gutta-Percha	140	0.45
Cortical Bone	13,700	0.3
Trabecular Bone	1370	0.3
Mta	11,760	0.314
Ribbond + Composite	23,600	0.32
Vita Enamic	30,000	0.23
IPS e-max ceramic	95,000	0.24

**Table 3:** Maximum tensile stress values observed for the structures involved in different models (MPa).

	Enamel	Dentin	Coronal Restoration	Core Structure	MTA	Cortical Bone	Spongy Bone
**Sound Tooth**	2.606	1.322				0.904	0.692
**Composite Resin**	4.130	3.891	2.330		0.412	2.151	0.995
**Fibre-Reinforced Composite**	4.577	3.841	2.686	0.951 (Fibre-Reinf. Core)	0.403	1.190	0.997
**Hybrid Ceramic Endocrown**	3.735	1.939	3.246		0.474	1.155	0.935
**LiSi Ceramic Endocrown**	3.675	1.763	3.300		0.404	1.105	0.909
**Endocore and Ceramic Crown**	3.621	1.578	3.478	1.475 (Endocore)	0.401	1.102	0.909

## Data Availability

No new data were created or analyzed in this study.

## References

[1] Cvek M. (1992). Prognosis of luxated non-vital maxillary incisors treated with calcium hydroxide and filled with gutta-percha: A retrospective clinical study. Endod. Dent. Traumatol..

[2] Murray P.E., Garcia-Godoy F., Hargreaves K.M. (2007). Regenerative endodontics: A review of current status and a call for action. J. Endod..

[3] AAE (2021). AAE Clinical Considerations for a Regenerative Procedure Revised 5/18/2021.pdf. https://www.aae.org/specialty/wp-content/uploads/sites/2/2021/08/ClinicalConsiderationsApprovedByREC062921.pdf.

[4] Balkaya H., Topçuoğlu H.S., Demirbuga S., Kafdağ Ö., Topçuoğlu G. (2022). Effect of different coronal restorations on the fracture resistance of teeth with simulated regenerative endodontic treatment: An in vitro study. Aust. Endod. J..

[5] Belli S., Erdemir A., Yildirim C. (2006). Reinforcement effect of polyethylene fibre in root-filled teeth: Comparison of two restoration techniques. Int. Endod. J..

[6] Shi R., Meng X., Feng R., Hong S., Hu C., Yang M., Jiang Y. (2022). Stress Distribution and Fracture Resistance of repairing Cracked Tooth with Fiber-reinforced Composites and Onlay. Aust. Endod. J..

[7] Dietschi D., Duc O., Krejci I., Sadan A. (2008). Biomechanical considerations for the restoration of endodontically treated teeth: A systematic review of the literature, Part II (Evaluation of fatigue behavior, interfaces, and in vivo studies). Quintessence Int..

[8] Vitale M.C., Caprioglio C., Martignone A., Marchesi U., Botticelli A.R. (2004). Combined technique with polyethylene fibers and composite resins in restoration of traumatized anterior teeth. Dent. Traumatol..

[9] Dotto L., Girotto L.P.S., Sousa Y.T.C.S., Pereira G.K.R., Bacchi A., Sarkis-Onofre R. (2024). Factors influencing the clinical performance of the restoration of endodontically treated teeth: An assessment of systematic reviews of clinical studies. J. Prosthet. Dent..

[10] Biacchi G.R., Mello B., Basting R.T. (2013). The endocrown: An alternative approach for restoring extensively damaged molars. J. Esthet. Restor. Dent..

[11] Fehrenbach J., de Soares J.L.S., Foly J.C.S.D.N., Miotti L.L., Münchow E.A. (2025). Mechanical performance of endocrown restorations in anterior teeth: A systematic review and network meta-analysis. Dent. Mater..

[12] Sedrez-Porto J.A., de Oliveira da Rosa W.L., da Silva A.F., Münchow E.A., Pereira-Cenci T. (2016). Endocrown restorations: A systematic review and meta-analysis. J. Dent..

[13] Qasim S.S.B., Zafar M.S., Niazi F.H., Alshahwan M., Omar H., Daood U. (2020). Functionally graded biomimetic biomaterials in dentistry: An evidence-based update. J. Biomater. Sci. Polym. Ed..

[14] Costa A., Xavier T., Noritomi P., Saavedra G., Borges A. (2014). The influence of elastic modulus of inlay materials on stress distribution and fracture of premolars. Oper. Dent..

[15] Ruse N., Sadoun M. (2014). Resin-composite blocks for dental CAD/CAM applications. J. Dent. Res..

[16] Miyazaki T., Hotta Y., Kunii J., Kuriyama S., Tamaki Y. (2009). A review of dental CAD/CAM: Current status and future perspectives from 20 years of experience. Dent. Mater. J..

[17] Eskitaşçioğlu M., Küçük O., Eskitaşçioğlu G., Eraslan O., Belli S. (2020). The Effect of Different Materials and Techniques on Stress Distribution in CAD/CAM Endocrowns. Strength. Mater..

[18] Chen J., Xu L. (1994). A finite element analysis of the human temporomandibular joint. J. Biomech. Eng..

[19] Asmussen E., Peutzfeldt A., Sahafi A. (2005). Finite element analysis of stresses in endodontically treated, dowel-restored teeth. J. Prosthet. Dent..

[20] Brito-Júnior M., Pereira R.D., Veríssimo C., Soares C.J., Faria-E-Silva A.L., Camilo C.C., Sousa-Neto M.D. (2014). Fracture resistance and stress distribution of simulated immature teeth after apexification with mineral trioxide aggregate. Int. Endod. J..

[21] Bucchi C., Marcé-Nogué J., Galler K.M., Widbiller M. (2019). Biomechanical performance of an immature maxillary central incisor after revitalization: A finite element analysis. Int. Endod. J..

[22] Anthrayose P., Nawal R.R., Yadav S., Talwar S., Yadav S. (2021). Effect of revascularisation and apexification procedures on biomechanical behaviour of immature maxillary central incisor teeth: A three-dimensional finite element analysis study. Clin. Oral. Investig..

[23] Belli S., Eraslan O., Eskitaşcıoğlu G. (2018). Effect of Different Treatment Options on Biomechanics of Immature Teeth: A Finite Element Stress Analysis Study. J. Endod..

[24] Demirel A., Bezgin T., Sarı Ş. (2021). Effects of Root Maturation and Thickness Variation in Coronal Mineral Trioxide Aggregate Plugs Under Traumatic Load on Stress Distribution in Regenerative Endodontic Procedures: A 3-dimensional Finite Element Analysis Study. J. Endod..

[25] Eram A., Zuber M., Keni L.G., Kalburgi S., Naik R., Bhandary S., Amin S., Badruddin I.A. (2020). Finite element analysis of immature teeth filled with MTA, Biodentine and Bioaggregate. Comput. Methods Programs Biomed..

[26] Jorquera G., Mahn E., Sanchez J.P., Berrera S., Prado M.J., Stange V.B. (2018). Hybrid ceramics in dentistry: A literature review. J. Clin. Res. Dent..

[27] Nelson S.J., Ash M.M. (1992). Wheeler’s Dental Anatomy, Physiology and Occlusion.

[28] Tada S., Stegaroiu R., Kitamura E., Miyakawa O., Kusakari H. (2003). Influence of implant design and bone quality on stress/strain distribution in bone around implants: A 3-dimensional finite element analysis. Int. J. Oral. Maxillofac. Implant..

[29] Desai S., Chandler N. (2009). The restoration of permanent immature anterior teeth, root filled using MTA: A review. J. Dent..

[30] Sorensen J.A., Martinoff J.T. (1984). Intracoronal reinforcement and coronal coverage: A study of endodontically treated teeth. J. Prosthet. Dent..

[31] Cramer N., Stansbury J., Bowman C. (2011). Recent advances and developments in composite dental restorative materials. J. Dent. Res..

[32] Eskitascioglu G., Belli S., Kalkan M. (2002). Evaluation of two post core systems using two different methods (fracture strength test and a finite elemental stress analysis). J. Endod..

[33] Zheng Z., Sun J., Jiang L., Wu Y., He J., Ruan W., Yan W. (2022). Influence of margin design and restorative material on the stress distribution of endocrowns: A 3D finite element analysis. BMC Oral. Health.

[34] Chen Z., Li Y., Deng X., Wang X. (2014). A novel computer-aided method to fabricate a custom one-piece glass fiber dowel-and-core based on digitized impression and crown preparation data. J. Prosthodont..

[35] Gulec L., Ulusoy N. (2017). Effect of endocrown restorations with different CAD/CAM materials: 3D finite element and weibull analyses. BioMed Res. Int..

[36] Vervack V., Johansson C., De Coster P., Fokkinga W., Papia E., Vandeweghe S. (2024). The fracture strength and the failure mode of lithium disilicate or resin nano ceramics as a crown, overlay, or endocrown restoration on endodontically treated teeth. J. Esthet. Restor. Dent..

[37] Shams A., Elsherbini M., Elsherbiny A.A., Özcan M., Sakrana A.A. (2022). Rehabilitation of severely-destructed endodontically treated premolar teeth with novel endocrown system: Biomechanical behavior assessment through 3D finite element and in vitro analyses. J. Mech. Behav. Biomed. Mater..

[38] Lin J., Zeng Q., Wei X., Zhao W., Cui M., Gu J., Lu J., Yang M., Ling J. (2017). Regenerative Endodontics Versus Apexification in Immature Permanent Teeth with Apical Periodontitis: A Prospective Randomized Controlled Study. J. Endod..

[39] Iosif L., Dimitriu B., Niţoi D.F., Amza O. (2023). Endodontic Dentistry: Analysis of Dentinal Stress and Strain Development during Shaping of Curved Root Canals. Healthcare.

[40] Ural Ç., Çağlayan E. (2021). A 3-dimensional finite element and in vitro analysis of endocrown restorations fabricated with different preparation designs and various restorative materials. J. Prosthet. Dent..

[41] Zhang Y., Lai H., Meng Q., Gong Q., Tong Z. (2022). The synergetic effect of pulp chamber extension depth and occlusal thickness on stress distribution of molar endocrowns: A 3-dimensional finite element analysis. J. Mater. Sci. Mater. Med..

[42] Huang Y., Fokkinga W.A., Zhang Q., Creugers N.H., Jiang Q. (2023). Biomechanical properties of different endocrown designs on endodontically treated teeth. J. Mech. Behav. Biomed. Mater..

[43] Belli S., Eraslan O., Eskitascioglu G. (2016). Effect of Root Filling on Stress Distribution in Premolars with Endodontic-Periodontal Lesion: A Finite Elemental Analysis Study. J. Endod..

